# Linalyl acetate prevents hypertension-related ischemic injury

**DOI:** 10.1371/journal.pone.0198082

**Published:** 2018-05-25

**Authors:** Yu Shan Hsieh, Soonho Kwon, Hui Su Lee, Geun Hee Seol

**Affiliations:** Department of Basic Nursing Science, School of Nursing, Korea University, Seoul, Republic of Korea; Loma Linda University School of Medicine, UNITED STATES

## Abstract

Ischemic stroke remains an important cause of disability and mortality. Hypertension is a critical risk factor for the development of ischemic stroke. Control of risk factors, including hypertension, is therefore important for the prevention of ischemic stroke. Linalyl acetate (LA) has been reported to have therapeutic effects in ischemic stroke by modulating intracellular Ca^2+^ concentration and having anti-oxidative properties. The preventive efficacy of LA has not yet been determined. This study therefore investigated the preventive efficacy of LA in rat aortas exposed to hypertension related-ischemic injury, and the mechanism of action of LA.Hypertension was induced *in vivo* following ischemic injury to the aorta induced by oxygen-glucose deprivation and reoxygenation *in vitro*. Effects of LA were assayed by western blotting, by determining concentrations of lactate dehydrogenase (LDH) and reactive oxygen species (ROS) and by vascular contractility assays. LA significantly reduced systolic blood pressure *in vivo*. *In vitro*, LA suppressed ischemic injury-induced expression of the nicotinamide adenine dinucleotide phosphate (NADPH) oxidase subunit p47^phox^, as well as ROS production, LDH release, and ROS-induced endothelial nitric oxide synthase suppression. These findings indicate that LA has anti-hypertensive properties that can prevent hypertension-related ischemic injury and can prevent NADPH oxidase-induced production of ROS.

## Introduction

Ischemic stroke is an important cause of mortality and disability, accounting for approximately 11% of alldeaths worldwide in 2016, according to theWorld Health Organization [1]. It has been reported that ischemic injury induces cell death through mechanisms that involve oxidative stress [2] and elevated intracellular calcium levels[3]. Confirming risk factors and evaluating underlying mechanisms are important for preventing ischemic stroke.Among the various risk factors for ischemic stroke, hypertension is the most important [4], with a previous study reporting that a considerable majority (69%) of ischemic stroke patients have a history of hypertension [5].

A trial of tissue plasminogen activator (tPA) by the National Institute of Neurological Disorders and Stroke (NINDS) provided evidence that thrombolytic therapy for ischemic stroke could improve neurological outcome, but the results were time sensitive. Unfortunately, because the symptoms of stroke are not detected early enough, more than 50% of patients arrive at the hospital too late (>3 hours) to be treated [6]. This delay reflects, at least in part, the difficulties in defining and identifying the symptoms of ischemic stroke. When an ischemic stroke occurs, it typically presents with a number of symptoms suggestive of stroke, including unilateral weakness or sudden numbness of the face or limbs, sudden dimness or loss of vision and speech, severe headache, or unexplained dizziness. However,these symptoms are also present in non-stroke patients [7]. Moreover, according to guidelines of the American Heart Association (AHA) and American Stroke Association, there are a number of contraindications to administration of intravenous (IV) tPA. These include hemorrhage, uncontrolled hypertension (systolic blood pressure [SBP] > 185 mm Hg or diastolic blood pressure [DBP]> 110 mm Hg), stroke or head trauma in the previous 3 months, thrombocytopenia and coagulopathy, among others [8]. Thus, despite the fact that the safety and efficacy of IV tPA has been repeatedly confirmed in previous studies, a disappointingly large number of patients with ischemic stroke still do not receive tPA treatment [9]. In addition to its numerous contraindications, tPA also has a critical side effect, namely hemorrhage [8]. As a result, it currently remains difficult to treat ischemic stroke and options are limited. Therefore, an aggressive preventive strategy is the optimal approach for decreasing ischemic injury,but better methods of prevention are necessary.

Linalyl acetate is the major constituent of the essential oil and extracts of *Citrus bergamiaRisso* (bergamot), *Lavandula angustifolia* (lavender) and *Salvia sclarea* (clary sage). It has been reported that numerous therapeutic effects, with recent research highlighting its inhibition of Ca^2+^influx in vascular endothelial cells[10], induction of vasorelaxation in nicotine-pretreated mouse aorta [11]. In another recent study, the linalyl acetate-rich essential oil of *Citrus bergamiaRisso*was shown to induce vasorelaxation through regulation of K^+^channels and subsequent inhibition of Ca^2+^ influx in mouse aortic vascular smooth muscle cells [12]. However, to date, there has been little research on the potential preventive effects of linalyl acetate, especially with respect to hypertension-related ischemic injury—a major cause of disability and mortality.

The aim of the present studywas to evaluate the mechanisms and preventive effectsof linalyl acetate on hypertension-related ischemic injury, with the ultimate goal of providing a new strategy for repair of ischemia-induced cell injury. To this end, we combined an *in vivo*model of hypertension (restraint stress plus nicotine treatment) that closely mimics the clinical situation[13], with an *ex vivo* model of ischemia, in which aortic tissue is exposed to oxygen-glucose deprivation and reoxygenation (OGD/R) conditions to induce subsequent ischemic injury.

## Materials and methods

### Experimental animals

Male 4-week-old Sprague-Dawley rats (n = 47) with a body weight of 100–110 g were obtained from Amteco Inc. (Korea) and housed at 22–23°C. Rats were allowed to move freely following a natural circadian rhythm (12 hours of light and 12 hours of darkness) and provided *ad libitum* access to food and water. All animal experimental procedures were approved by theInstitutional Animal Care and Use Committee in Korea University (KUIACUC-2016-153) and were in accord with the Guide for the Care and Use of Laboratory Animals published by the US National Institutes of Health (NIH Publication No. 85–23; revised 1996).For the *in vivo* component of the experimental model, rats were divided randomly into five groups: Normotension (n = 13), Hypertension (n = 15), Hypertension+25 mg/kg linalyl acetate [LA25] (n = 6), Hypertension+50 mg/kg linalyl acetate [LA50] (n = 7), and Hypertension+100 mg/kg linalyl acetate [LA100] (n = 6), and Hypertension+15 mg/kg acetylsalicylic acid (ASA) [A] (n = 6) as a positive control group.

### Hypertensive ischemic injury model

Hypertension was induced by combined immobilization stress (2 h/d in a restraint cage) and intraperitoneal injection of nicotine (0.8 mg/kg/d) for 21 days and nicotine (3 mg/kg/d) on 22 day. This was followed on day 22 by pretreatment with different doses of linalyl acetate (25, 50, 100 mg/kg) or ASA (15 mg/kg)[14], delivered as a bolus dose. Rats were then allowed to move freely for 60 minutes to enable drug absorption before sacrificing.Blood pressure (BP) variation was assessed by monitoring BP once weekly using a tail cuff andpulse transducer (AD Instruments, Sydney, Australia).Rats in the Normotensive group were not exposed to immobilization stress, and received an intraperitoneal injection of 0.9% normal saline daily instead of nicotine. On day 22, rats in this group were injected with polyethylene glycol-200 (PEG) instead of linalyl acetate or ASA.

At the end of the combined immobilization stress/nicotine treatment regimen, rats were anesthetized with isoflurane and then sacrificed by cervical dislocation. The aorta was then collected and cleaned of connective tissue. Aorta samples were divided into seven groups: Normotension(n = 7), Normotension+OGD (n = 6), Hypertension (n = 7), Hypertension+OGD (n = 8), Hypertension+OGD+LA25 (n = 6), Hypertension+OGD+LA50 (n = 7), Hypertension+OGD+LA100 (n = 6)and Hypertension+OGD+A (n = 6). Aortas in each group were immersed in Dulbecco's Modified Eagle’s medium, with or without glucose (as indicated), in an Airlock Anaerobic Chamber (Coy Lab Products, USA). Ischemic injury was induced *ex vivo* by oxygen-glucose deprivation, produced by exposure to a gas mixture containing 5% CO_2_ and 10% H_2_ (partial pressure of oxygen balanced below 2 mmHg) in the absence of glucose for 45 minutes, followed by reoxygenation for 30 minutes with saturated 95% O_2_ and 5% CO_2_in a 37°C incubator; the non-OGD group was exposed to reoxygenation for 75 minutes at 37°C [15].

**Fig 1:**
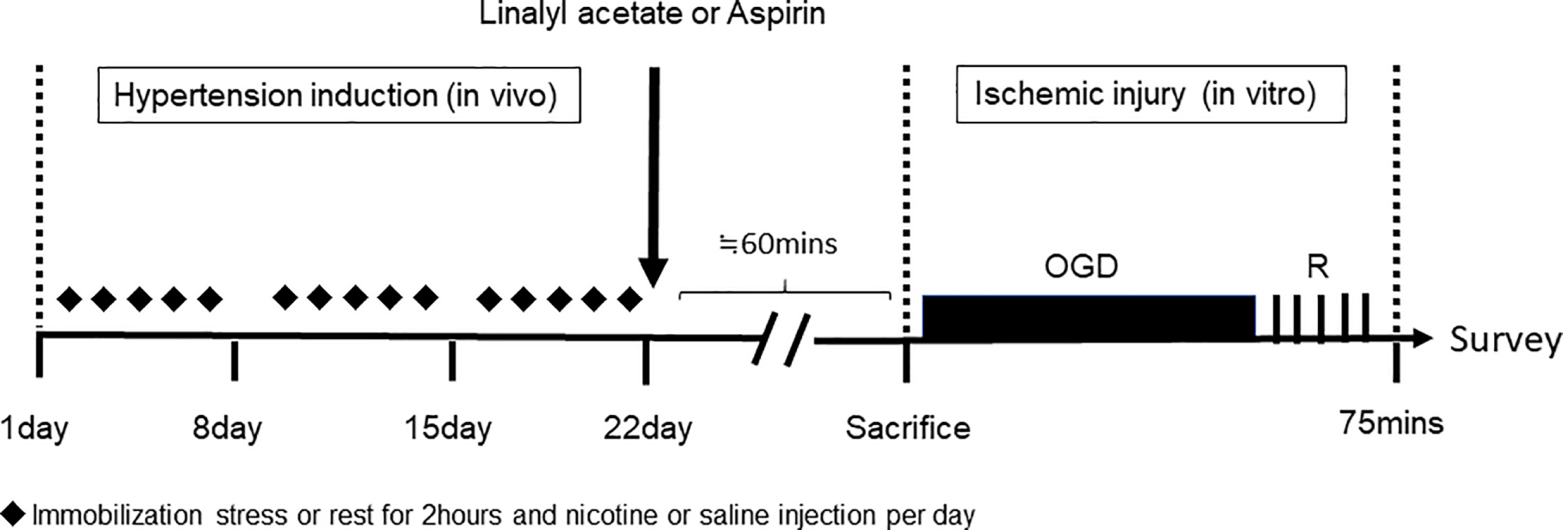
Experimental design of the hypertension + OGDmodel in rats. Hypertension was induced in vivo from day 1 to day 22. After injecting linalyl acetate, ASA or PEG (45–60 minutes), rats were sacrificed, and aortas were removed. Ischemic injury was then induced by exposing aortic segments to OGD (45 minutes) and reoxygenation (30 minutes), after which tissues were used in experiments.

The hypertension-ischemic injury model, created by combining *in vivo* hypertension-induction and *ex vivo* ischemic injury exposure (Fig 1), allowed the preventive effects of linalyl acetate to be assessed.

### Isolated rat aortic rings and contractility assay

Rat aortas were cut into 2-mm segments and placed in an organ bath (620M; Danish Myo Technology, Denmark) or 96-well tissue culture plate for OGD and reoxygenation treatment using an Airlock Anaerobic Chamber (Coy Lab Products). At the end of OGD (or control) treatments, aortic rings were mounted on a myograph, and then washed three times with fresh Krebs solution (118.3 mM NaCl, 25 mM NaHCO_3_, 1.22 mM KH_2_PO_4_, 1.2 mM MgCl_2_, 4.78 mM KCl, 2.5 mM CaCl_2_, and 11.1 mM glucose). Aortic segments were then equilibrated for 50 minutes under 1.0 g of resting tension. Isotonic contractions of artery segments were recorded with a mechano-transducer connected to a computer. Vasoconstriction was evoked by adding 10 μM phenylephrine (PE); at the end of experiments, aortas were fully relaxed by adding 10 μM sodium nitroprusside (SNP) or acetylcholine (ACh).

### Reactive oxygen species (ROS) assays

Endothelial cells of rat abdominal aortas were isolated, mounted on glass slides, and fixed with 4% paraformaldehyde. Slides were incubated for 45 minutes at 37°C with 5 μM2,7-dichlorofluorescin diacetate (DCFH-DA) (Abcam, UK) to probe for ROS, followed by incubation for 5 minutes at 37°C with Hoechst 33342 (Sigma Chemical Co., USA) to counterstain nuclei. DCFH-DA fluorescence was detected using a Nikon DS-Ri2 fluorescence microscope (Nikon, Japan) at a magnification of 400X by exciting at a wavelength of 485 nm and collecting emitted fluorescence at 535 nm. Fluorescence intensity was quantified using NIS Elements image analysis software (Nikon, Japan) andImage J software (National Institutes of Health, USA), and results are presented as mean relative fluorescence intensity (RFI) values ± S.E.M.

### Lactate dehydrogenase (LDH) assays

Cell viability in all treatment groups was quantified by measured LDH released into the rat aorta tissue culture medium. The level of LDH released into the tissue culture medium was assayed using a CytoTox 96 Non-Radioactive Cytotoxicity Assay kit (Promega Co., USA) according to the manufacturer’s instructions. Changes in absorbance at 340 nm were measured using a microplate ELISA reader (BMG Labtech, Germany), and results are presented as mean optical density (OD) values ± S.E.M.

### Western blotting

After treatments, whole and endothelium-denudedrat aortic tissue werehomogenized, and the protein concentration in each lysate was adjusted to 30 μg/25 μl for Western blotting. Samples were separated by sodium dodecyl sulfate-polyacrylamide gel electrophoresis (SDS-PAGE) on 10% gels, and then electrophoretically transferred to a nitrocellulose membrane. The membrane was first incubated overnight with primary anti-eNOS (1:300; Santa Cruz Biotechnology, USA), anti-NADPH (nicotinamide adenine dinucleotide phosphate) oxidase subunit p47^phox^ (1:500; Thermo-Fisher, USA) and anti-glyceraldehyde-3-phosphate dehydrogenase (GADPH) (1:1500; Santa Cruz Biotechnology) antibodies, and then with the appropriate secondary antibody for 60 minutes at room temperature. Immunoreactive proteins were detected using enhanced chemiluminescence regents (Bio-Rad, USA). Proteins were quantified by densitometric analysis of pixel density using Image J software (National Institutes of Health).

### Chemicals

Dulbecco's Modified Eagle's mediumwithout glucose, nicotine ((–) nicotine hydrogen tartrate salt), linalyl acetate, acetylsalicylic acid, and PEG-200 were purchased from Sigma ChemicalCompany.

### Statistical analysis

Results of tests are presented as mean values ± S.E.M. Differences in mean values among groupswere analyzed using a one-way analysis of variance (ANOVA) with LSD post hoc test using SPSS Statistics 22 version (IBM, USA). *p*-values lessthan 0.05 were considered statistically significant.

## Results

### Preventive effect of linalyl acetate against hypertension- related ischemic injury

To evaluate the preventive effects of linalyl acetate, we treated rats after inducing hypertension by combined restrain stress and nicotine treatment, but before *ex vivo*exposure of arteries to ischemic injury. Using this combination model, we evaluated the therapeutic effects of linalyl acetate on hypertension and its preventive effect on ischemic injury (Fig 1).Beginning on day 8, SBP was significantly higher in the hypertensive than in the normotensive group (*p* = 0.003) (S1 Fig).

### Blood pressure regulatory effects of linalyl acetate in the rat model of hypertension

**Fig 2:**
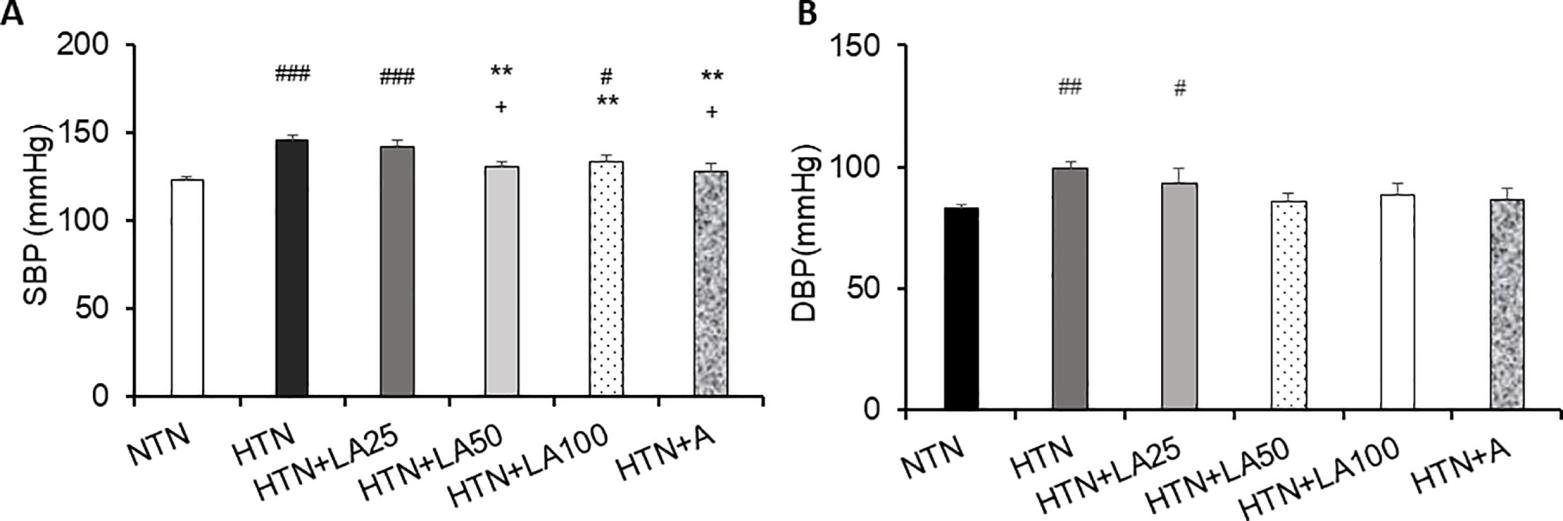
Differences in (A) SBP and (B) DBP among groups on day 22 after linalyl acetate or PEG injection. Results are presented as means ± SEM (#p< 0.05, ## p < 0.01, ### p < 0.001 compared with the normotensive group; ** p< 0.01 compared with the hypertensive group; + p< 0.01 compared with the hypertension + 25 mg/kg linalyl acetate group). NTN, normotension; HTN, hypertension; HTN+LA25, hypertension+25 mg/kg linalyl acetate; HTN+LA50, hypertension+50 mg/kg linalyl acetate; HTN+LA100, hypertension+100 mg/kg linalyl acetate, HTN+A, hypertension+15 mg/kg ASA.

To determine the effects of different doses oflinalyl acetate on blood pressure, we measured systolic blood pressure (SBP) and diastolic blood pressure (DBP) 45 minutes afterinjection of linalyl acetate, acetylsalicylic acid or PEG (control). We found that hypertension-induced increases in SBP were significantly decreased by higher doses oflinalyl acetate, with significant reductions in SBP observed at 50 mg/kg and 100 mg/kg linalyl acetate (*p* = 0.001). Treatment with 15 mg/kg ASA also reduced SBP (*p* = 0.001) (Fig 2A). At doses of 50 mg/kg and 100 mg/kg, linalyl acetate injection also showed a trend toward decreased DBP, but the reductions were not significant (Fig 2B)

### Inhibitory effect of linalyl acetate on p47^phox^expression in the hypertension-ischemia injury model

**Fig 3:**
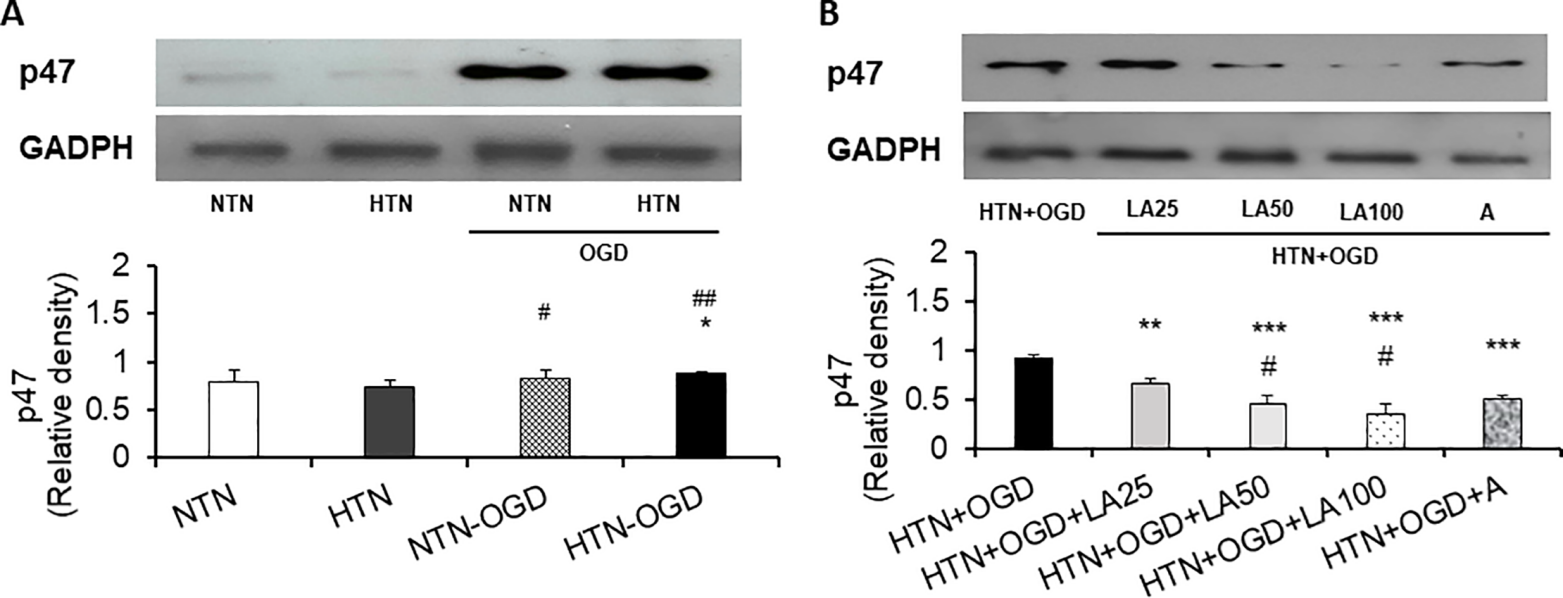
Effects of linalyl acetate on ischemic injury-induced p47phox expression. (A) Evaluation of p47phox expression levels after hypertension and OGD/R exposure. NTN, normotension; HTN, hypertension; NTN+OGD, normotension+ OGD; HTN+OGD, hypertension + OGD. Results are presented as means ± SEM (#p< 0.05, ## p< 0.01 compared with the NTN group; *p< 0.05 compared with the HTN group).(B) Inhibitory effects of linalyl acetate on p47phox expression in the HTN+OGD group. HTN+OGD+LA25, hypertension+OGD+25 mg/kg linalyl acetate; HTN+OGD+LA50, hypertension+OGD+50 mg/kg linalyl acetate; HTN+OGD+LA100, hypertension+OGD+100 mg/kg linalyl acetate; HTN+OGD+ A, hypertension+OGD+15 mg/kg ASA. Results are presented as means ± SEM (**p< 0.01, ***p < 0.001 compared with the HTN+OGD group; # p< 0.01 compared with the HTN+OGD+LA25 group).

Utilizing Western blot analysis, we investigated hypertensive ischemic injury-induced changes in p47^phox^ expression and corresponding inhibitory effects of linalyl acetate. These analyses showed that hypertensive ischemic injury induced p47^phox^ overexpression, measured as pixel density, in the hypertension-only group (*p* = 0.017) (Fig 3A).These findings, however, were not observed in endothelium-denuded aorta tissue of the hypertension-only group (S2 Fig). Notably, linalyl acetate induced a dose-dependent decrease in p47^phox^ expression, reducing p47^phox^ intensity at 25, 50 and 100 mg/kg compared with a control value (*p* < 0.001), 15 mg/kg ASA also reducing p47^phox^ intensitycompared with the control value (*p* < 0.001) (Fig 3B).

### Inhibitory effects of linalyl acetate on ROS production and ROS-induced cell damage in a hypertension-ischemia injury model

**Fig 4:**
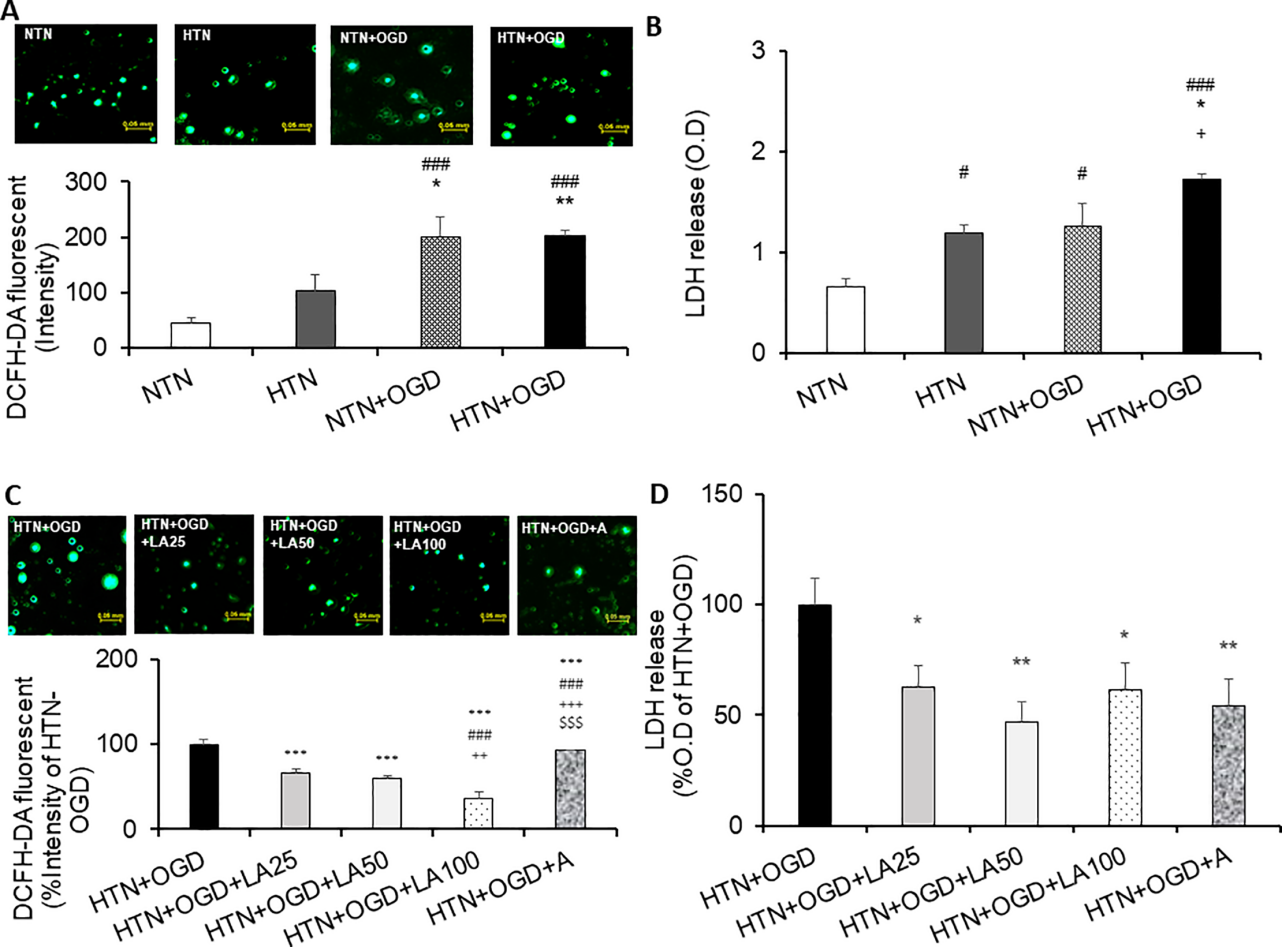
Effects of linalyl acetate on ischemic injury-induced ROS production and LDH release. (A) Evaluation of injury-induced ROS production and (B) LDH release after hypertension and OGD/R exposure. NTN, normotension; HTN, hypertension; NTN+OGD, normotension + OGD; HTN+OGD, hypertension + OGD. Results are presented as means ± SEM (#p< 0.05, ### p < 0.001 compared with the NTN group; *p < 0.05 compared with the HTN group). (C) Inhibitory effects of linalyl acetate on ischemic injury-induced ROS production and (D) LDH release in the HTN+OGD group. HTN+OGD+LA25, hypertension+OGD+25 mg/kg linalyl acetate; HTN+OGD+LA50, hypertension+OGD+50 mg/kg linalyl acetate; HTN+OGD+LA100, hypertension+OGD+100 mg/kg linalyl acetate; HTN+OGD+A, hypertension+OGD+15 mg/kg ASA. Results are presented as means ± SEM (*p < 0.05, **p < 0.01, ***p< 0.001 compared with the HTN+OGD group; #p < 0.05 compared with the HTN+OGD+LA25 group; ++ p < 0.01, +++p < 0.001 compared with the HTN+OGD+LA50 group; $ $ $p < 0.001 compared with the HTN+OGD+LA100 group).

Using DCFH-DA, a widely used fluorescent probe for detecting intracellular ROS, and measurements of extracellular release of LDH release, a cytosolic enzyme used as an indicator of cellular toxicity and damage, we monitored ROS production and ROS-induced cell damage in our hypertension-ischemic injury model. These analyses showed that ROS production, expressed as relative fluorescence units (RFIs), was significantly increased by hypertensive-ischemic injury (*p* = 0.004) compared with hypertension alone (Fig 4A). As shown in Fig 4B, LDH release, measured as optical density (OD) units, was similarly increased in the hypertensive-ischemic injury group (*p* = 0.025) compared with the hypertensive-only group. Importantly, the enhanced ROS production induced by hypertension-ischemic injury was significantly reduced by linalyl acetate, which exerted a dose-dependent attenuation in ROS levels. At 25, 50 and 100 mg/kg, linalyl acetate decreased ROS levels by 33.9%, 40.4% and 63.1%, respectively, while 15 mg/kg ASA treatment decreased ROS levels by 6.7%, compared with that in the hypertensive-ischemic injury group (*p* < 0.001) (Fig 4C). As shown in Fig 4D, hypertensive-ischemic injury-induced LDH release was also significantly decreased by linalyl acetate at doses of 25 mg/kg (37.3%,*p* = 0.029), 50 mg/kg (52.9%, *p* = 0.002) and 100 mg/kg (38.6%, *P* = 0.032), as well as by 15 mg/kg ASA (45.8%, *p* = 0.007).

### Effect of linalyl acetate on ROS-induced eNOSSuppression in a hypertension-ischemia injury model

**Fig 5:**
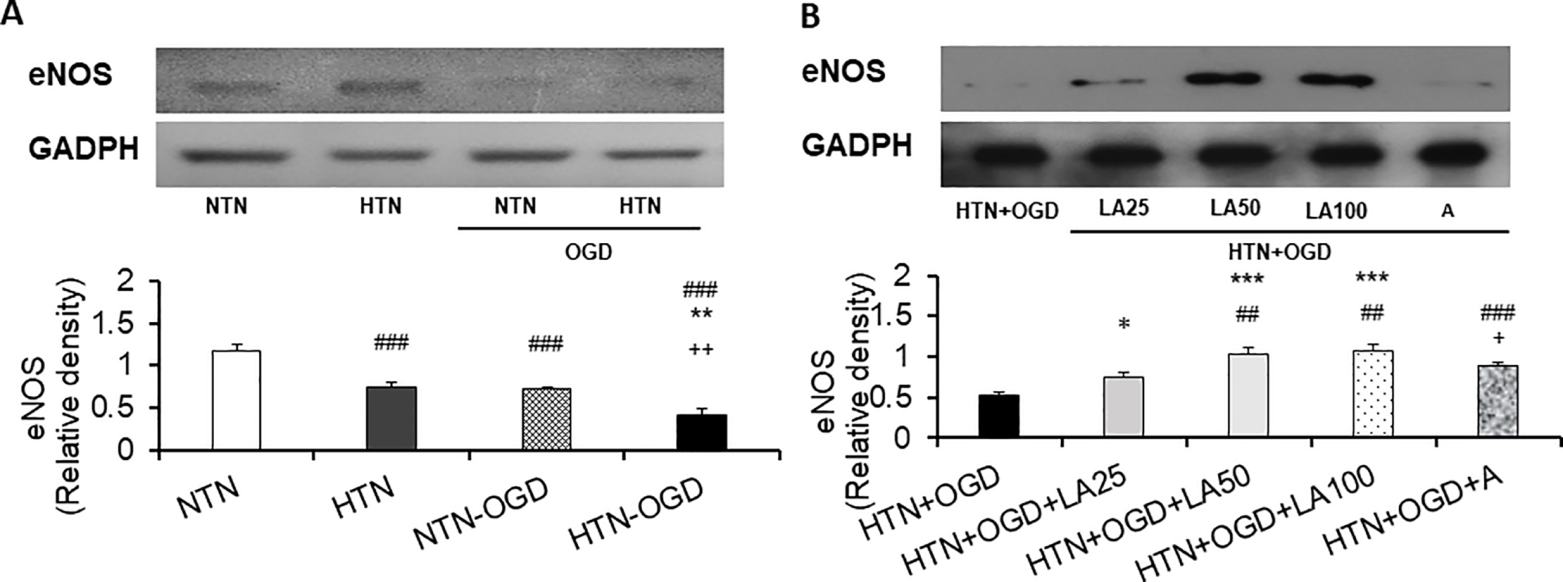
Effects of linalyl acetate on ROS-induced suppression of eNOS expression. (A) Evaluation of ROS-induced eNOS suppression.NTN, normotension; HTN, hypertension; NTN+OGD, normotension+OGD; HTN+OGD, hypertension+OGD. Results are presented as means ± SEM (###p < 0.001 compared with the NTN group; **p< 0.01 compared with the HTN group; ++p< 0.01 compared with the NTN+OGD group). (B)Preventive effects of linalyl acetate on ROS-induced eNOS suppression. HTN+OGD+LA25, hypertension+OGD+25 mg/kg linalyl acetate; HTN+OGD+LA50, hypertension+OGD+50mg/kg linalyl acetate; HTN+OGD+LA100, hypertension+OGD+100 mg/kg linalyl acetate; HTN+OGD+A, hypertension+OGD+15 mg/kg ASA. Results are presented as means ± SEM (*p < 0.05,***p < 0.001 compared with the HTN+OGD group; ##p< 0.01, ### p < 0.001 compared with the HTN+OGD+LA25 group; +p < 0.05 compared with the HTN+OGD+LA50 group).

Next, we used Western blotting to investigate changes inthe expression of endothelial nitric oxide synthase (eNOS). These analyses indicated that hypertensive-ischemic injury-induced ROS production significantly suppressed eNOS expression compared with that in the hypertension-only group (*p* = 0.002) (Fig 5A). Our data further indicate that linalyl acetate significantly reversed ROS-induced eNOS suppression, increasing pixel density values at doses of 25, 50, and 100 mg/kg, respectively (*p* < 0.001) (Fig 5B). Similarly, 15 mg/kg ASA treatment was also associated with increased pixel density values(*p* < 0.001).

### VasorelaxingEffects of linalyl acetate in a hypertension-ischemia injury model

**Fig 6:**
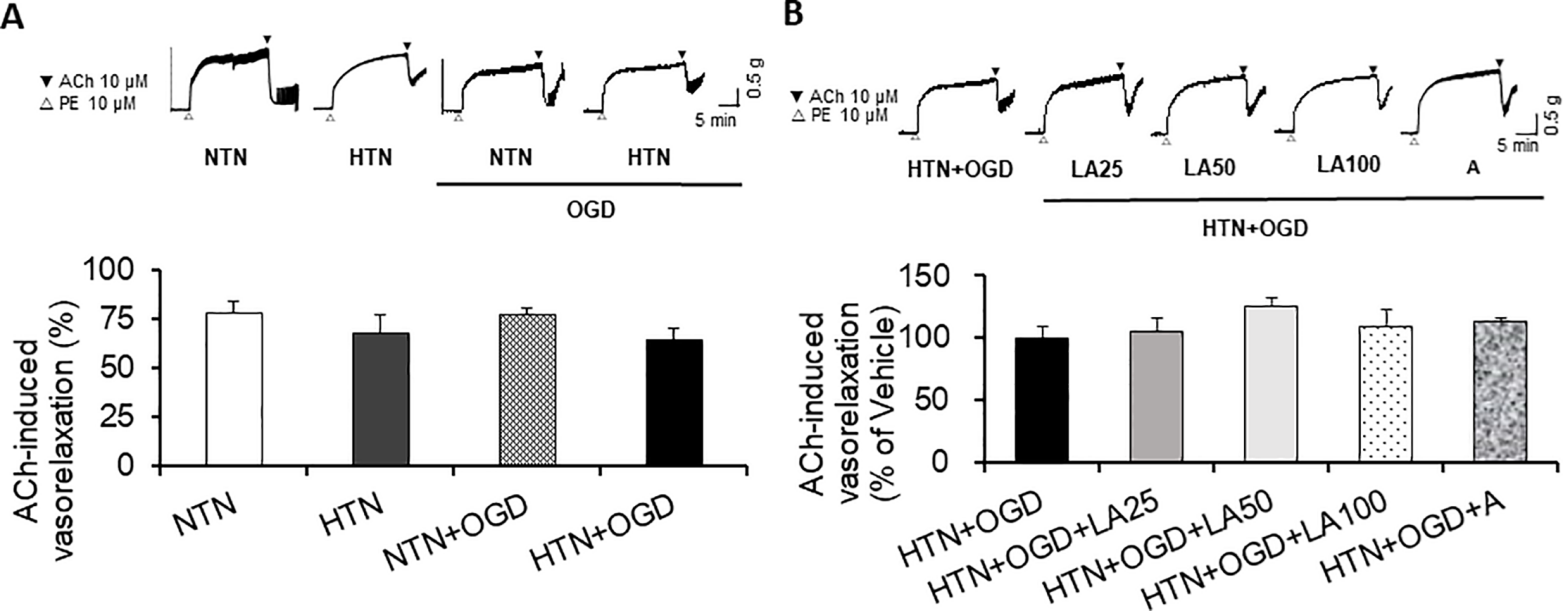
Effects of hypertension and ischemic injury. (A) On ACh-induced, endothelium-dependent relaxation (trace) and relaxation rate. NTN, normotension; HTN, hypertension; NTN+OGD, normotension+OGD; HTN+OGD, hypertension+OGD. Results are presented as means ± SEM.(B) Effects of linalyl acetate on ischemic injury-relatedACh-induced, endothelium-dependent relaxation (trace) and relaxation rate.HTN+OGD+LA25, hypertension+OGD+25 mg/kg linalyl acetate; HTN+OGD+LA50, hypertension+OGD+50 mg/kg linalyl acetate; HTN+OGD+LA100, hypertension+OGD+100 mg/kg linalyl acetate; HTN+OGD+ A, hypertension+OGD+15 mg/kg ASA.

Finally, we tested ACh-induced vasorelaxation (which is mediated by NO) in aortic segments from hypertension-ischemia model rats that had been preconstricted with the sympathomimetic amine, phenylephrine.These experiments showed that PE-induced vasoconstriction was significantly reduced in the normotensive ischemic-injury group (*p* = 0.023) compared with that in the uninjured normotensive group, whereas ischemic injury had no effect on PE-induced vasoconstriction in the hypertensive group (data not shown). In contrast to the results obtained for PE-induced vasoconstriction, ACh-induced vasorelaxation was not significantly altered by ischemic injury in either the normotensive group or hypertensive group (Fig 6A).These experiments further showed that linalyl acetate had no significant effect on PE-induced vasoconstriction at 25, 50, or 100 mg/kg, and that ASA at 15 mg/kg also had no significant effect, compared with the hypertension+OGD group (data not shown). Although there was a modest trend toward increased ACh-induced vasorelaxation with linalyl acetate pretreatment at 25, 50, or 100 mg/kg or with 15 mg/kg ASA pretreatment, compared with the hypertension + OGD group, these differences did not reach statistical significance (Fig 6B).

## Discussion

Ischemic stroke is a leading cause of disability and mortality. Early delivery of tPA—3 to 4.5 hours after ischemic stroke onset—to patients that satisfy eligibility requirements to receive the treatment has been demonstrated to reduce disability [16]. However, tPA has been shown to only recanalize the occluded internal carotid artery and middle cerebral artery by approximately 27%[17]. Moreover, in EXTEND-IA (EmergencyNeurological Deficits with Intra-Arterial) trials, tPA was only shown to succeed in recanalizing the arterial occlusion in somewhat more than 15% of patients [18]. To date, treatment of ischemic stroke remains difficult and therapeutic options are limited. Therefore, prevention—notably through controlling hypertension, one of the major risk factors—is considered a promising alternative strategy for reducing the incidence, recurrence, associated disability and mortality rates of ischemic stroke [19].

Oxygen-glucose deprivation (OGD) and middle cerebral artery occlusion (MCAO) models are frequently used for research on ischemic stroke *in vitro*, *ex vivo* and *in vivo*. In the OGD/R model, tissues or cells cultured without glucose and oxygen are transferred into an environment without oxygen for hours to induce ischemic cell injury, after which the injured cells are returned to a normal environment containing glucose and oxygen for a period to mimic the reperfusion associated with an ischemic stroke [20].Similarly, in the MCAO model, ischemic injury is induced by unilaterally occluding the MCA of animals with a suture for a period, after which the suture is removed to allow reperfusion[21]. Both OGD and MCAO similarly induce expression of ischemic injury-related signals [22], but MCAO can induce irreversible brain injury. In a previous study,a 1-hour MCAO period was shown to cause severe brain injury involving more than 50% of the ipsilateral hemisphere volume[23].These attributes of the MCAO model make it unfavorable for use in evaluating preventive effects in the current study. In addition, MCAO may increase the risk of mortality in animal models.Notably, our hypertensive-ischemic injury combination model allowed us to simultaneously evaluate therapeutic effects on hypertension and preventive effects on ischemic injury.

It has been reported that smoking and chronic stress are significantly associated with hypertension and stroke incidence [24, 25].Stress is considered a cause for many people to smoke, but smoking can also cause stress and anxiety. Thus, many people become trapped in a vicious cycle of stress and smoking[26]. A recent report showed that, in the United States, almost 19% of patients who had cardiovascular disease were also cigarette smokers [27]. Therefore, to create a hypertension-ischemic injury model that approached the actual situation, we used a combination of immobilization stress and nicotine—the “active” and addictive ingredient of cigarettes.

ASA,or aspirin, is an antithrombotic agent that is widely prescribed to prevent ischemic stroke or myocardial infarction. It has also proven efficacious in the prevention of ischemic stroke [28] and cardiovascular disease in clinical trials [29]. In addition, ASA has been found to inhibit Ca^2+^ influx in rat inferior colliculus neurons[30], and to decrease ROS and ROS-induced eNOS in human vein endothelial cells[31]. Therefore, we used ASA as a positive control medicine to evaluate the preventive effect of linalyl acetate in a hypertension-ischemic injury rat model.

**Fig 7:**
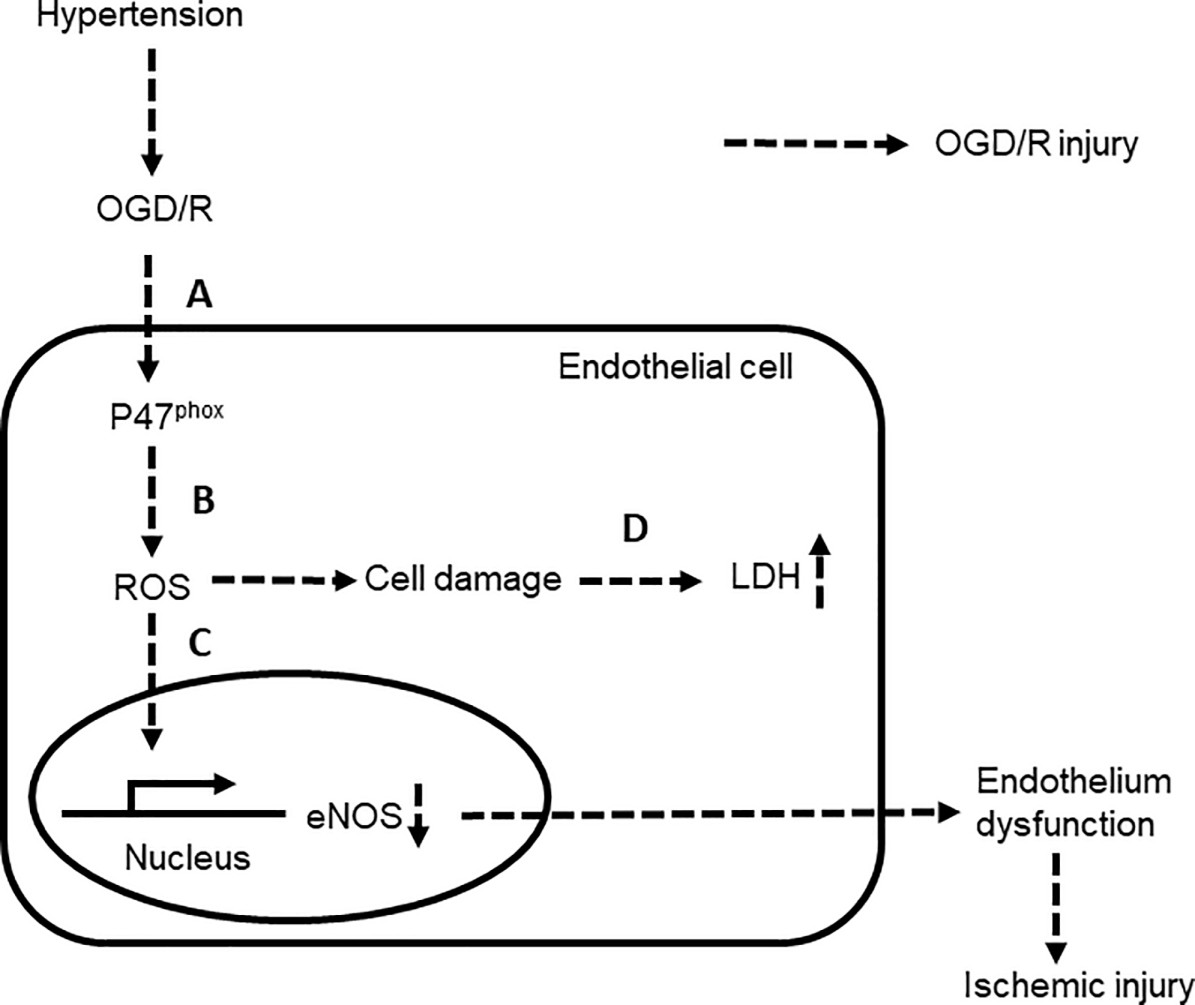
Mechanisms underlying the preventive effects of linalyl acetate on ischemic injury in endothelial cells. (A) Suppression of phosphorylation and activation of the NADPH oxidase p47phox subunit, and (B) the consequent decrease in ROS production, (C) thereby preventing ROS-induced eNOS suppression, and (D) decreasing oxidative stress-induced LDH release.

In a previous study, linalyl acetate was reported to increase NO production in the aorta of mice pretreated with nicotine [11]. Notably, NO generated by eNOS acts as an intracellular regulator of systemic vascular resistance that ultimately affects blood pressure in humans[32]. In this study, we found that linalyl acetate had a preventive effect on the suppression of eNOS, partially restoring eNOS protein levels in a rat model of hypertensive ischemic injury (Fig 7C). We further confirmed that 50 mg/kg and 100 mg/kg doses of linalyl acetate decreased the hypertension-induced elevation of SBP in this rat model. Collectively, these results suggest that linalyl acetate exerts a cardioprotective effect.

Intracellular ROS production is considered to play an important role in inducing hypertensive and ischemic injury [33] and subsequently enhance LDH release [34]. NADPH oxidase (NOX), a transmembrane protein complexthat transports electrons across cell membranes, is a major producer of intracellular ROS. Subunits of the NOX complex include the membrane-bound proteins p22^phox^ and gp91^phox^; the cytosolic proteins p47^phox^, p67^phox^, p40^phox^; and the small G-protein Rac1. NOX is activated by phosphorylation of the cytosolic subunit p47^phox^[35], which results in translocation of subunits to the cell membrane, formation of the active NOX complex, and an increase in ROS production. Thus, inhibition of NOX may be beneficial in ischemic injury[36]. ROS can reduce expression of eNOS, an enzyme that plays a critical role in the maintenance of vascular tone and vascular pressure through regulation of NO, thereby leading to ischemic injury-induced endothelial celldysfunction. In the present study, pretreatment with linalyl acetate prior to ischemic injury exposure indeed decreased ROS production through inactivation of the NOX subunit p47^phox^(Fig 7A). ROS-induced eNOS suppression and LDH release were also prevented by linalyl acetate pre-treatment. These results show that linalyl acetate is capable of preventing ROS production through its anti-p47^phox^ effects.

Previous studies have shown that hypoxia/ischemia induces vasorelaxation, reflecting activation of membrane K_ATP_ channels in arterial smooth muscle cells and inhibition of Ca^2+^ influx [37]. Accordingly, thedecrease of PE-induced vasoconstriction was observed in theischemic injury-only group, but not in the hypertensive-ischemic injury group.The cytosolic Ca^2+^ concentration plays a key role in the regulation of vasoconstriction [38], and if increased abnormally by ROS, it may disrupt vasoconstriction and vasorelaxation [39]. However, the molecular mechanisms underlying the cardiovascular effects of linalyl acetate are still poorly understood. Therefore, our future experiments will focus on further evaluating changes in molecular markers in this hypertension-ischemic injury model.

In conclusion, the results presented here show that linalyl acetate exerts excellent preventive effects on hypertension-related ischemic injury through its anti-hypertensive efficacy and modulation of eNOS expression. These results contribute to our understanding of the mechanism responsible for the preventive effects of linalyl acetate, but more importantly, may lead to a new strategy for preventing hypertensive ischemic strokes in a clinical setting.

## Supporting information

S1 Fig SBP and DBP in the normotension and hypertension groups. SBP and DBP were measured before test and on days 1, 8, 15, and 22.Results are presented as means ± SEM (^#^*p*< 0.05, ^##^*p*< 0.01, ^###^*p*< 0.001 compared with the normotensive group. ** *p* < 0.01 compared with the hypertensive group; ^+^ *p* < 0.01 compared with the hypertension + 25 mg/kg linalyl acetate group).
(TIF)

S2 Fig Effect of linalyl acetate on p47^phox^ expression in endothelium-denuded aorta tissue of the hypertension and hypertension+OGD groups. (TIF)

## References

[1] WHO. WHO methods and data sources for country‐level causes of death 2000‐2015 http://www.who.int/healthinfo/global_burden_disease/GlobalCOD_method_2000_2015.pdf?ua=120162016

[2] Martin LJ, Brambrink AM, Price AC, Kaiser A, Agnew DM, Ichord RN, et al Neuronal death in newborn striatum after hypoxia-ischemia is necrosis and evolves with oxidative stress. Neurobiology of disease. 2000;7(3):169–91. doi: 10.1006/nbdi.2000.0282 10860783 10.1006/nbdi.2000.0282

[3] Kristian T, Siesjo BK. Calcium in ischemic cell death. Stroke. 1998;29(3):705–18. 9506616 10.1161/01.str.29.3.705

[4] Hu G, Sarti C, Jousilahti P, Peltonen M, Qiao Q, Antikainen R, et al The impact of history of hypertension and type 2 diabetes at baseline on the incidence of stroke and stroke mortality. Stroke. 2005;36(12):2538–43. doi: 10.1161/01.STR.0000190894.30964.75 16282538 10.1161/01.STR.0000190894.30964.75

[5] Qureshi AI, Ezzeddine MA, Nasar A, Suri MF, Kirmani JF, Hussein HM, et al Prevalence of elevated blood pressure in 563,704 adult patients with stroke presenting to the ED in the United States. The American journal of emergency medicine. 2007;25(1):32–8. doi: 10.1016/j.ajem.2006.07.008 17157679 10.1016/j.ajem.2006.07.008 PMC2443694

[6] Tilley BC, Lyden PD, Brott TG, Lu M, Levine SR, Welch KM. Total quality improvement method for reduction of delays between emergency department admission and treatment of acute ischemic stroke. The National Institute of Neurological Disorders and Stroke rt-PA Stroke Study Group. Archives of neurology. 1997;54(12):1466–74. 9400355 10.1001/archneur.1997.00550240020008

[7] Schroeder EB, Rosamond WD, Morris DL, Evenson KR, Hinn AR. Determinants of use of emergency medical services in a population with stroke symptoms: the Second Delay in Accessing Stroke Healthcare (DASH II) Study. Stroke. 2000;31(11):2591–6. 11062280 10.1161/01.str.31.11.2591

[8] Jauch EC, Saver JL, Adams HP Jr., Bruno A, Connors JJ, Demaerschalk BM, et al Guidelines for the early management of patients with acute ischemic stroke: a guideline for healthcare professionals from the American Heart Association/American Stroke Association. Stroke. 2013;44(3):870–947. doi: 10.1161/STR.0b013e318284056a 23370205 10.1161/STR.0b013e318284056a

[9] Fugate JE, Rabinstein AA. Absolute and Relative Contraindications to IV rt-PA for Acute Ischemic Stroke. The Neurohospitalist. 2015;5(3):110–21. doi: 10.1177/1941874415578532 26288669 10.1177/1941874415578532 PMC4530420

[10] You JH, Kang P, Min SS, Seol GH. Bergamot essential oil differentially modulates intracellular Ca2+ levels in vascular endothelial and smooth muscle cells: a new finding seen with fura-2. Journal of cardiovascular pharmacology. 2013;61(4):324–8. doi: 10.1097/FJC.0b013e3182834681 23288200 10.1097/FJC.0b013e3182834681

[11] Kim JR, Kang P, Lee HS, Kim KY, Seol GH. Cardiovascular effects of linalyl acetate in acute nicotine exposure. Environmental Health and Preventive Medicine. 2017;22(1):42 doi: 10.1186/s12199-017-0651-6 29165169 10.1186/s12199-017-0651-6 PMC5664431

[12] Kang P, Suh SH, Min SS, Seol GH. The essential oil of Citrus bergamia Risso induces vasorelaxation of the mouse aorta by activating K(+) channels and inhibiting Ca(2+) influx. The Journal of pharmacy and pharmacology. 2013;65(5):745–9. doi: 10.1111/jphp.12031 23600392 10.1111/jphp.12031

[13] Han AY, Lee YS, Kwon S, Lee HS, Lee KW, Seol GH. Codonopsis lanceolata extract prevents hypertension in rats. Phytomedicine: international journal of phytotherapy and phytopharmacology. 2018;39:119–24. 29433673 10.1016/j.phymed.2017.12.028

[14] Khayyam N, Thavendiranathan P, Carmichael FJ, Kus B, Jay V, Burnham WM. Neuroprotective effects of acetylsalicylic acid in an animal model of focal brain ischemia. Neuroreport. 1999;10(2):371–4. 10203337 10.1097/00001756-199902050-00029

[15] Kolesarova M, Pavel J, Lukacova N, Kolesar D, Marsala J. Effect of ischemia in vivo and oxygen-glucose deprivation in vitro on NOS pools in the spinal cord: comparative study. Cellular and molecular neurobiology. 2006;26(7–8):1281–94. doi: 10.1007/s10571-006-9032-1 16691443 10.1007/s10571-006-9032-1 PMC11520762

[16] Lees KR, Bluhmki E, von Kummer R, Brott TG, Toni D, Grotta JC, et al Time to treatment with intravenous alteplase and outcome in stroke: an updated pooled analysis of ECASS, ATLANTIS, NINDS, and EPITHET trials. Lancet. 2010;375(9727):1695–703. doi: 10.1016/S0140-6736(10)60491-6 20472172 10.1016/S0140-6736(10)60491-6

[17] Saqqur M, Uchino K, Demchuk AM, Molina CA, Garami Z, Calleja S, et al Site of arterial occlusion identified by transcranial Doppler predicts the response to intravenous thrombolysis for stroke. Stroke. 2007;38(3):948–54. doi: 10.1161/01.STR.0000257304.21967.ba 17290031 10.1161/01.STR.0000257304.21967.ba

[18] Campbell BC, Mitchell PJ, Yan B, Parsons MW, Christensen S, Churilov L, et al A multicenter, randomized, controlled study to investigate EXtending the time for Thrombolysis in Emergency Neurological Deficits with Intra-Arterial therapy (EXTEND-IA). International journal of stroke: official journal of the International Stroke Society. 2014;9(1):126–32. 24207098 10.1111/ijs.12206

[19] Joseph LN, Babikian VL, Allen NC, Winter MR. Risk factor modification in stroke prevention: the experience of a stroke clinic. Stroke. 1999;30(1):16–20. 9880382 10.1161/01.str.30.1.16

[20] Lu Q, Wainwright MS, Harris VA, Aggarwal S, Hou Y, Rau T, et al Increased NADPH oxidase-derived superoxide is involved in the neuronal cell death induced by hypoxia-ischemia in neonatal hippocampal slice cultures. Free radical biology & medicine. 2012;53(5):1139–51. 22728269 10.1016/j.freeradbiomed.2012.06.012 PMC3527086

[21] Paulson JR, Yang T, Selvaraj PK, Mdzinarishvili A, Van der Schyf CJ, Klein J, et al Nicotine exacerbates brain edema during in vitro and in vivo focal ischemic conditions. The Journal of pharmacology and experimental therapeutics. 2010;332(2):371–9. doi: 10.1124/jpet.109.157776 19889792 10.1124/jpet.109.157776 PMC2812118

[22] Yao S, Tang B, Li G, Fan R, Cao F. miR-455 inhibits neuronal cell death by targeting TRAF3 in cerebral ischemic stroke. Neuropsychiatric disease and treatment. 2016;12:3083–92. doi: 10.2147/NDT.S121183 27980410 10.2147/NDT.S121183 PMC5147416

[23] Kim ES, Ahn SY, Im GH, Sung DK, Park YR, Choi SH, et al Human umbilical cord blood-derived mesenchymal stem cell transplantation attenuates severe brain injury by permanent middle cerebral artery occlusion in newborn rats. Pediatric research. 2012;72(3):277–84. doi: 10.1038/pr.2012.71 22669296 10.1038/pr.2012.71

[24] Wolf PA, D'Agostino RB, Kannel WB, Bonita R, Belanger AJ. Cigarette smoking as a risk factor for stroke. The Framingham Study. Jama. 1988;259(7):1025–9. 3339799

[25] Sparrenberger F, Cichelero FT, Ascoli AM, Fonseca FP, Weiss G, Berwanger O, et al Does psychosocial stress cause hypertension? A systematic review of observational studies. Journal of human hypertension. 2009;23(1):12–9. doi: 10.1038/jhh.2008.74 18615099 10.1038/jhh.2008.74

[26] McCrae RR, Costa PT, Bossé R. Anxiety, extraversion and smoking. British Journal of Social and Clinical Psychology. 1978;17(3):269–73. 687883 10.1111/j.2044-8260.1978.tb00277.x

[27] Go AS, Mozaffarian D, Roger VL, Benjamin EJ, Berry JD, Borden WB, et al Executive Summary: Heart Disease and Stroke Statistics—2013 Update. A Report From the American Heart Association. 2013;127(1):143–52. 10.1161/CIR.0b013e318282ab8f 23283859

[28] Antithrombotic Trialists C. Aspirin in the primary and secondary prevention of vascular disease: collaborative meta-analysis of individual participant data from randomised trials. Lancet. 2009;373(9678):1849–60. doi: 10.1016/S0140-6736(09)60503-1 19482214 10.1016/S0140-6736(09)60503-1 PMC2715005

[29] Ittaman SV, VanWormer JJ, Rezkalla SH. The Role of Aspirin in the Prevention of Cardiovascular Disease. Clinical Medicine & Research. 2014;12(3–4):147–54. 24573704 10.3121/cmr.2013.1197 PMC4317158

[30] Liu Y, Li X, Ma C, Liu J, Lu H. Salicylate blocks L-type calcium channels in rat inferior colliculus neurons. Hearing research. 2005;205(1–2):271–6. doi: 10.1016/j.heares.2005.03.028 15953536 10.1016/j.heares.2005.03.028

[31] Ou HC, Lee WJ, Wu CM, Chen JF, Sheu WH. Aspirin prevents resistin-induced endothelial dysfunction by modulating AMPK, ROS, and Akt/eNOS signaling. Journal of vascular surgery. 2012;55(4):1104–15. doi: 10.1016/j.jvs.2011.10.011 22244860 10.1016/j.jvs.2011.10.011

[32] Stamler JS, Loh E, Roddy MA, Currie KE, Creager MA. Nitric oxide regulates basal systemic and pulmonary vascular resistance in healthy humans. Circulation. 1994;89(5):2035–40. 7514109 10.1161/01.cir.89.5.2035

[33] Rodrigo R, Fernandez-Gajardo R, Gutierrez R, Matamala JM, Carrasco R, Miranda-Merchak A, et al Oxidative stress and pathophysiology of ischemic stroke: novel therapeutic opportunities. CNS & neurological disorders drug targets. 2013;12(5):698–714. 23469845 10.2174/1871527311312050015

[34] Bagchi D, Bagchi M, Hassoun EA, Stohs SJ. In vitro and in vivo generation of reactive oxygen species, DNA damage and lactate dehydrogenase leakage by selected pesticides. Toxicology. 1995;104(1–3):129–40. 8560491 10.1016/0300-483x(95)03156-a

[35] Bedard K, Krause KH. The NOX family of ROS-generating NADPH oxidases: physiology and pathophysiology. Physiological reviews. 2007;87(1):245–313. doi: 10.1152/physrev.00044.2005 17237347 10.1152/physrev.00044.2005

[36] Walder CE, Green SP, Darbonne WC, Mathias J, Rae J, Dinauer MC, et al Ischemic stroke injury is reduced in mice lacking a functional NADPH oxidase. Stroke. 1997;28(11):2252–8. 9368573 10.1161/01.str.28.11.2252

[37] Gasser R, Brussee H, Wallner M, Kickenweiz E, Grisold M, Rotman B, et al Current views on mechanisms of vasodilation in response to ischemia and hypoxia. International Journal of Angiology. 1993;2(1):22.

[38] Nilius B, Droogmans G. Ion channels and their functional role in vascular endothelium. Physiological reviews. 2001;81(4):1415–59. doi: 10.1152/physrev.2001.81.4.1415 11581493 10.1152/physrev.2001.81.4.1415

[39] Wang YW, Zhang JH, Yu Y, Yu J, Huang L. Inhibition of Store-Operated Calcium Entry Protects Endothelial Progenitor Cells from H2O2-Induced Apoptosis. Biomolecules & therapeutics. 2016;24(4):371–9. 27169819 10.4062/biomolther.2015.130 PMC4930280

