# Supplementary figures and images for: Linalyl acetate prevents hypertension-related ischemic injury
Source: PLoS One. 2018 May 25;13(5):e0198082. doi: 10.1371/journal.pone.0198082 (PMC5969747; doi:10.1371/journal.pone.0198082)

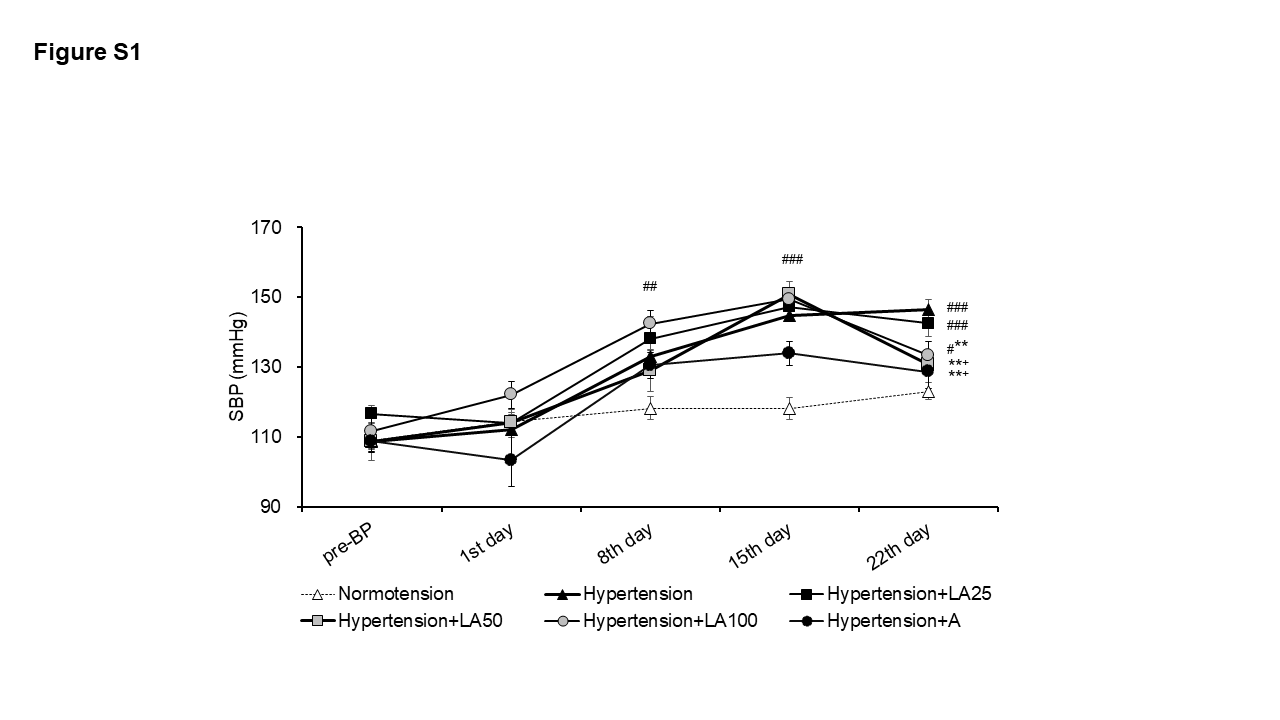

Supplement: S1 Fig — SBP and DBP were measured before test and on days 1, 8, 15, and 22.Results are presented as means ± SEM (#p< 0.05, ##p< 0.01, ###p< 0.001 compared with the normotensive group. ** p < 0.01 compared with the hypertensive group; + p < 0.01 compared with the hypertension + 25 mg/kg linalyl acetate group). (TIF) [file pone.0198082.s001.TIF]

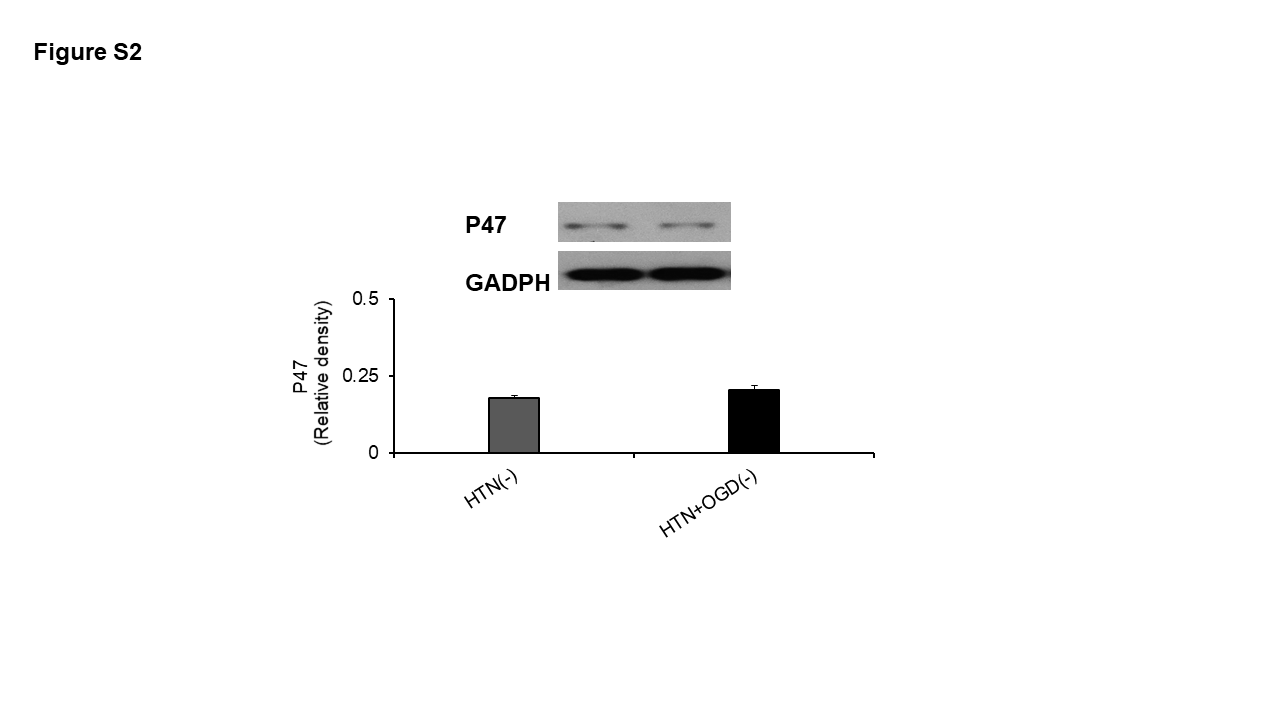

Supplement: S2 Fig — (TIF) [file pone.0198082.s002.TIF]
